# Web-Based Cognitive Behavioral Therapy for Depression Among Homebound Older Adults: Development and Usability Study

**DOI:** 10.2196/47691

**Published:** 2023-09-19

**Authors:** Xiaoling Xiang, Jay Kayser, Samson Ash, Chuxuan Zheng, Yihang Sun, Addie Weaver, Ruth Dunkle, James A Blackburn, Alex Halavanau, Jia Xue, Joseph A Himle

**Affiliations:** 1 School of Social Work University of Michigan-Ann Arbor Ann Arbor, MI United States; 2 Department of Psychology University of Michigan-Ann Arbor Ann Arbor, MI United States; 3 School of Social Work Columbia University New York City, NY United States; 4 SLAC National Accelerator Laboratory Stanford University Menlo Park, CA United States; 5 Factor-Inwentash Faculty of Social Work University of Toronto Toronto, ON Canada; 6 Faculty of Information University of Toronto Toronto, ON Canada

**Keywords:** internet-based cognitive behavioral therapy, usability, geriatric depression, community-engaged research, web-based, geriatrics, geriatric, depression, psychotherapy, mental health, older adults, older adult, cognitive behavioral therapy, CBT, design, development, community, user centered design, digital health, aging, old age, digital mental health, web-based health, internet

## Abstract

**Background:**

Homebound older adults are a high-risk group for depression. However, many of them face barriers to accessing evidence-supported mental health treatments. Digital mental health interventions can potentially improve treatment access, but few web-based interventions are explicitly tailored for depression in older adults.

**Objective:**

This paper describes the development process of Empower@Home, a web-delivered intervention for depression in homebound older adults that is based on cognitive behavioral therapy, and reports on the outcomes of usability studies.

**Methods:**

Empower@Home was developed in collaboration with community agencies, stakeholders, and older adults, guided by user-centered design principles. User needs were assessed through secondary data analysis, demographic and health profiles from administrative data, and interviews and surveys of community partners. A comparative usability evaluation was conducted with 10 older adults to assess the usability of Empower@Home compared to 2 similar programs. Field testing was conducted with 4 end users to detect additional usability issues.

**Results:**

Feedback and recommendations from community partners heavily influenced the content and design of Empower@Home. The intervention consists of 9 sessions, including psychoeducation and an introduction to cognitive behavioral therapy skills and tools through short video clips, in-session exercises, an animated storyline, and weekly out-of-session home practice. A printed workbook accompanies the web-based lessons. In comparative usability testing (N=10), Empower@Home received a System Usability Scale score of 78 (SD 7.4), which was significantly higher than the 2 comparator programs (*t*_9_=3.28; *P*=.005 and *t*_9_=2.78; *P*=.011). Most participants, 80% (n=8), preferred Empower@Home over the comparators. In the longitudinal field test (n=4), all participants reported liking the program procedures and feeling confident in performing program-related tasks. The single-subject line graph showed an overall downward trend in their depression scores over time, offering an encouraging indication of the intervention’s potential effects.

**Conclusions:**

Collaboration with community stakeholders and careful consideration of potential implementation issues during the design process can result in more usable, engaging, and effective digital mental health interventions.

## Introduction

Being homebound is often linked with socioeconomic disadvantages, including low income, racial minority status, and high levels of disability. Studies have shown that half of homebound older adults exhibit clinically significant symptoms of depression, with 14% meeting the criteria for current major depression [1,2]. This starkly contrasts the 2% prevalence of major depression in nonhomebound older adults [3]. When left untreated or insufficiently treated, depression can reduce quality of life and increase hospitalizations and early mortality [4]. Despite the availability of evidence-based treatments, traditional office-based services often remain out of reach for homebound older adults due to access barriers such as cost, transportation, and stigma [5]. Insurance coverage options for minor depression are also limited, and few mental health clinicians have received specialized training in working with older adults. It is crucial to find innovative ways to provide evidence-supported psychosocial treatments that are both accessible and cost-effective while reducing reliance on highly trained professionals and ensuring scalability.

Digital mental health interventions (DMHIs) are behavioral and psychological intervention strategies that use technology, such as websites, mobile apps, and other mobile devices, to improve mental health. Internet-based cognitive behavioral therapy, or iCBT, is one of the most studied DMHIs. iCBT is an automated psychotherapy based on cognitive behavioral therapy (CBT) principles delivered via dedicated websites or apps [6]. Patients receive psychoeducational materials through a web platform or a dedicated app and are exposed to the same core components as conventional CBT (eg, behavioral activation and cognitive restructuring). At a reduced marginal cost, iCBT can be used repeatedly with different patients without losing its therapeutic power, making it particularly useful for reducing health disparities in underresourced settings [7]. Studies have shown that iCBT is as effective as face-to-face CBT in treating depression in mixed-age samples [8]. Emerging evidence also supports the potential benefits of iCBT in older adults, including those with a heightened risk of depression [9,10].

However, most iCBT programs have not been specifically tailored to meet the needs of older adults, with only a few exceptions [11-14]. For older adults, this includes procedural and content modifications to CBT that address differences in thinking styles and age-related adjustment [15]. In addition, the user interface (UI) in web-delivered interventions may need to be adjusted to fit the preferences, needs, and capabilities of older adults [16]. Furthermore, we are unaware of such programs in the US market tailored explicitly for homebound older adults. Generic DMHIs can benefit older adults, but we have found that those with complex interfaces often result in low adherence and engagement, limited effects, and a myriad of usability issues among low-income, homebound older adults [10,17]. These individuals are typically less tech-savvy and more sensitive to usability problems.

Our team developed Empower@Home, a web-based psychosocial depression intervention explicitly designed for homebound older adults, to address the shortage of DMHIs tailored to this high-need and underserved population. Empower@Home is a 9-session iCBT program that aims to prevent and reduce the symptoms of depression. The target population is homebound adults aged >60 years (ie, those with mobility difficulties). The intervention development process involved significant stakeholder input and user-centered design principles and occurred alongside academic-community partnership development. In this paper, we describe the process of developing Empower@Home, report on its feasibility and usability evaluation outcomes, and discuss its implications for designing DMHIs that are attuned to the needs of individuals and the characteristics of implementation settings.

## Methods

### The Empower@Home Intervention

Empower@Home includes 9 web-based lessons, each featuring didactic content, in-session exercises, motivational quotes, and an engaging animated story driven by human characters. Table 1 presents an overview of each session. Each lesson is presented in brief videos (less than 2 minutes) to lessen cognitive load (Multimedia Appendix 1). The lessons are arranged in a specific sequence, and each concludes with instructions for home practice. During home practices, users apply the skills they have learned using various program tools. These tools focus on fundamental CBT skills and are grouped into categories: doing tools for behavioral activation and problem-solving, thinking tools for cognitive restructuring, feeling tools for relaxation and mood monitoring, and communication tools for fostering effective communication. In addition, participants do a mood self-check by filling out the Patient Health Questionnaire-9 (PHQ-9) in every other session (sessions 1, 3, 5, 7, and 9) to track their symptoms [18].

**Table 1:** Empower@Home session-by-session overview^a^.

Session	Session content	CBT^b^ elements	Home practice
Session 1: Ready, Set… Go!	Session 1 orients the user to the program, delivers psychoeducation about depression and aging, and gently introduces CBT in jargon-free language. The session also includes content to motivate the user to engage with the program and introduces BA^c^, which is referred to as a doing tool.	Psychoeducation Doing tools: activity monitoring	Activity monitoring form
Session 2: Doing Tools	Session 2 is a continued exploration of BA using the value-based BA approach. Major in-session activities include reviewing the activity monitoring form from the last session, charting the depression downward spiral, filling out the values and activities inventory, and creating the “my desired activities” master list. The first mindfulness exercise––Body Scan––is also introduced in this session.	Doing tools: value-based behavioral activation; activity scheduling Feeling tools: body scan	Body scan and activity scheduling
Session 3: Working with Barriers	Session 3 continues the focus on BA skills by addressing common barriers to BA for older adults. Major in-session activities include practicing breaking things down into small steps, completing the “my desired activities” master list by adding the names of supportive people, and turning “you statements” into “I statements.” This session also discusses the characteristics of effective communication.	Doing tools: break down tasks; activity scheduling Communication tools: effective communication and I statements	Activity scheduling
Session 4: Keep Doing	Session 4 continues to address common barriers to BA, including unhelpful thoughts (eg, “I can’t do anything”) and physical barriers to doing things. The user learns about adaptive behaviors and behavioral modification methods. Issues like independence and getting help are also discussed. Major in-session exercises include adaptive behavior quizzes, identifying inner strengths, and making adjustments to the “my desired activities” master list. The second mindfulness exercise, called mind-calming, is introduced.	Doing tools: adaptive behaviors Thinking tools: unhelpful thoughts related to BA Feeling tools: mind-calming exercise	Mind-calming exercise and activity scheduling
Session 5: Problem Solving	Session 5 provides a 5-step problem-solving technique. The user follows along to practice the technique using their own problem, concluding in an action plan. The second communication tool––active listening––is introduced.	Doing tools: 5-step problem-solving Communication tools: active listening	Problem-solving and activity scheduling
Session 6: Unhelpful Thinking	Session 6 is the first of two sessions on cognitive restructuring—another core CBT skill. The user learns about common unhelpful thinking patterns and is asked to identify them in case stories and reflect on their experience. Core beliefs are also introduced.	Thinking tools: ABC model; identify unhelpful thoughts and core beliefs.	Thought record and activity scheduling
Session 7: Thinking Tools	Session 7 is the second session on cognitive restructuring and moves from identifying thinking errors to challenging them. The “7-column thought record” is introduced to continue tracking thinking errors and practicing challenging methods.	Thinking tools: challenge unhelpful thoughts	Thought record and activity scheduling
Session 8: Feeling Tools	Session 8 discusses various forms of self-care and addresses physical activity and nutrition. The second half of the session introduces mindfulness and walks the users through a guided breathing exercise. Another mindfulness exercise––the senses exercise––is also introduced.	Feeling tools: self-care; mindfulness	Breathing exercise and activity scheduling
Session 9: Putting It All Together	As the last session of the program, Session 9 reviews the core techniques taught and addresses relapse prevention. The user follows along to create their empowerment guide. The user also learns about other treatment options like medication, one-on-one therapy, and other therapies.	Program review and relapse prevention	Relapse prevention plan

^a^The list of sessions and content presented in this table is the most updated version and is being tested in an ongoing pilot randomized controlled trial.

^b^CBT: cognitive behavioral therapy.

^c^BA: behavioral activation.

The web-based sessions are enriched with an animated case story series featuring a 74-year-old homebound woman named Jackie (Multimedia Appendix 2). The animated story series is embedded within each session, similar to a television show episode, to reinforce and further illustrate the application of core CBT skills and techniques. The inclusion of an animated case story aligns with persuasive design and uses entertainment to elicit strong emotional responses [19].

The web-based program is accompanied by a printed user workbook in large print, containing session summaries, in-session exercises, directions and forms for home practices, inspirational quotes, and wellness resources (Multimedia Appendix 3). Typically, iCBT programs offer web-based worksheets or workbooks. However, we provided a printed workbook considering the target population’s likely familiarity with print media and the commonly reported issues regarding text entry from other iCBT studies involving older adults [20].

Using agile, state-of-the-art development processes, we built the web platform as a custom learning management system and made it accessible across various devices. The main interface features large buttons, icons with text descriptions, high-contrast color schemes, and intuitive navigation, all of which follow the current best practices for creating age-friendly UIs [16]. In addition, we designed a provider dashboard that allows providers to review patients’ progress, enabling them to readily access easily digestible data for quality improvement and evaluation purposes (Multimedia Appendix 4).

### Design Overview

The intervention development followed a user-centered design process and involved 3 iterative steps: elicitation, design, and usability testing, as outlined by Kruzan et al [21]. In the elicitation phase, we drew on multiple data sources to inform our understanding of end user needs, preferences, and requirements.

In the design phase, we used a co-design approach to create the treatment manual, case stories, and media design in collaboration with community stakeholders. These stakeholders included older adult advisors, geriatric mental health professionals, and aging services providers from 14 community organizations in Michigan. Over 100-plus meetings, the team iteratively refined the scripts of web-based sessions, voice-over actors, animated character designs, media designs, workbook designs, and other program elements based on stakeholder input. The core research team worked with a user experience designer to develop a wireframe, followed by a low-fidelity prototype. This prototype was tested by researchers and older adult stakeholders and refined based on their feedback.

In the usability testing phase, the ready-to-release version of Empower@Home underwent a heuristic evaluation by researchers and User Experience designers, followed by an in-home comparative usability study involving think-aloud exercises and longitudinal field testing with end users. Design needs and refinements to both the treatment manual and the web interfaces were made after each evaluation.

We assembled a multidisciplinary team to support our design activities, including mental health researchers, gerontologists, human-computer interaction researchers, user experience designers, web developers, and community stakeholders. We used recommended eHealth development strategies, such as heavy stakeholder participation, an iterative design process, continuous evaluations, and integration of implementation issues and concerns into the design process [22].

### Elicitation

Multiple data sources informed our understanding of the needs, preferences, and requirements of end users and the community settings that are likely to implement the intervention. First, we reanalyzed qualitative data from 21 homebound older adults who had participated in our prior study on a generic iCBT program. We applied a deductive coding approach guided by the efficiency model of support [23] to identify issues surrounding usability (ease of use), engagement (motivation), fit (meeting user’s needs), knowledge (how to use a tool), and implementation (how to apply the tool into user’s life). The procedures of the prior study are detailed elsewhere [17]. We edited the transcripts, preserving only text relevant to our research question. A priori codes were developed based on the efficiency model of support and codes from our prior work. New codes were inductively added as we analyzed the transcripts. After the codes were finalized, we then identified patterns and categorized the initial codes into a smaller number of groups, themes, and concepts.

Our second data source was summary statistics of participants in Michigan’s 1915(c) MI Choice Waiver program, generously shared by colleagues. This program caters to our target demographic of low-income homebound older adults.

In addition, we established partnerships with social service agencies that serve many homebound older adults. We conducted semistructured interviews and a web-based survey with these organizations to identify potential barriers to implementing DMHIs at both the provider and organization levels. Each organization was profiled, and common barriers to implementation were identified through a descriptive analysis of the survey data and a text analysis of the qualitative data.

### Design

The design process primarily used co-design meetings and passive storyboarding techniques. Given that CBT is considered gold standard psychotherapy for depression, the core session elements were informed by widely used, evidence-supported CBT manuals [24-27]. In our regular meetings with community stakeholders, we shared each session’s content by reading it aloud, thus soliciting immediate feedback and stimulating discussions on potential improvements to both content and delivery. Revisions, guided by meeting notes, were typically integrated within a day or two. Frequent meetings were held with stakeholders from various organizations each week. The revised content would then be tested in subsequent meetings with different stakeholder groups, an iterative process that continued until the core development team was satisfied that all feedback had been addressed. This labor-intensive co-design process served to not only craft the intervention but also foster partnerships with community organizations. Given that these activities unfolded amidst the COVID-19 pandemic, individual co-design meetings over Zoom (Zoom Video Communications, Inc) were deemed the most viable way to garner feedback from each stakeholder group.

While social service providers, many of whom are social workers, significantly influenced the psychoeducational content of the program, the development of the character-driven animated story heavily relied on input from homebound older adults. These senior advisors contributed to weekly small group discussions, guiding the development of characters, plot, script, and visual design. Each story episode was role-played during these meetings to obtain feedback on tone, dialogue, and alignment with the educational objectives of the sessions. Following each meeting, the core development team convened for a debrief, and revisions were promptly implemented. This iterative process of presenting revised scripts and design elements continued until no further feedback was forthcoming.

### Usability Testing

#### In-Home Comparative Usability Evaluation

In a single in-home session, we conducted a comparative usability evaluation of 3 different DMHI programs, including Beating the Blues, MoodGym, and Empower@Home. Ten homebound older adults were recruited through UMHealthResearch, a volunteer registry maintained by the University of Michigan. Participants were eligible if they were at least 60 years old and homebound. Homebound status was broadly defined as self-reported difficulty with outdoor mobility or receipt of in-home care or home-delivered meals. Prior computer experience was not required. A diagnosis of depression or elevated depressive symptoms was not required.

Beating the Blues was selected due to a solid body of evidence supporting its effectiveness [28,29] and core features shared with Empower@Home, including using a mix of audio and video-based content to teach CBT techniques, case examples, in-session exercises, and homework practice. Unlike Empower@Home, Beating the Blues uses real actors in its case examples. MoodGym, which has no audio or video material, was also included in the evaluation. MoodGym is a popular iCBT program and has been shown to reduce depressive symptoms [30]. Of the 3 programs, Empower@Home was the only program specifically designed for older adults.

The order of trials was based on a predetermined random sequence to reduce the influence of learning effects. Participants spent up to 20 minutes per program and were asked to think aloud as they performed tasks such as reviewing information on the web pages, advancing to the next page, navigating between program components, and completing in-session exercises. After each trial, participants completed the System Usability Scale (SUS), followed by open-ended questions to probe their experiences, likes, and dislikes. After participants tried all 3 programs, they were asked to select their favorite program and explain their choice.

The SUS is a 10-item scale commonly used in evaluating the usability of websites, software, and other human-machine systems [31]. Scoring the SUS involves reverse coding the negatively worded statements and summing up all 10 item scores. The sum was then multiplied by 2.5 so that the total SUS score ranged from 0 (very poor perceived usability) to 100 (excellent perceived usability) in 2.5-point increments. A SUS score above 68 is considered above average. The SUS is a valid measure to compare systems [32] and has excellent internal consistency in the study sample (Cronbach α=.92).

Data were collected at each participant’s home, and all participants engaged with the 3 programs on a 10.5-inch tablet provided by the study team. Participants’ interactions with the screen were recorded using a screen recording app. One researcher with user experience design training took detailed notes of participants’ interactions with each program.

Descriptive statistics were conducted to describe the study sample and the SUS scores. Paired sample 2-tailed *t* tests were used to compare the SUS scores of Empower@Home and those of the 2 comparator programs. The user experience designer coded the responses to open-ended questions, field notes, and observations and generated a 1-page report, which aided in the interpretation of the SUS scores and their differences.

#### Longitudinal Field Testing

We conducted longitudinal field testing with low-income homebound older adults through a small open-pilot trial. Participants in the field test were recruited via community partner agency referrals. Participants needed to (1) read and speak English, (2) be at least 60 years old, and (3) have at least mild depressive symptoms at screening (≥5 on the PHQ-9) [18]. Individuals were ineligible if they had (1) probable dementia based on the Blessed Orientation, Memory, and Concentration test (score>9) [33]; (2) elevated suicide risk based on the Columbia-Suicide Severity Rating Scale [34]; (3) a terminal illness or unstable physical health conditions; or (4) severe vision impairment. Device ownership, prior computer use, or internet access were not required.

A 10.5-inch tablet with cellular data was provided to participants without technology access. Participants were given 10 weeks to complete the program with minimal support from project staff in the form of a brief weekly check-in that typically lasted between 5 and 10 minutes. Participants were invited to complete a short survey before the start of the program and then again at the end of the 10-week trial. Participants also completed up to 5 in-app assessments based on the PHQ-9.

Descriptive statistics were conducted to describe the study sample. Given the small sample size, inferential statistics were not computed. Instead, a single-subject line graph was used to visualize the changes in the PHQ-9 scores over time. Furthermore, participants’ feedback and field notes were coded and consolidated to uncover any additional usability concerns.

### Ethics Approval

The University of Michigan Institutional Review Board approved the usability study and the field-testing study (HUM00207612). Written informed consent was obtained at the start of the home visit for the usability study. Verbal informed consent was obtained from each participant before the start of the program for the field-testing study.

## Results

### Elicitation

Secondary data analysis of qualitative data from our previous project revealed various user needs and potential failure points to address. The key problems identified were usability issues that frustrated older participants. These included hard-to-read text, small clickable areas, perplexing navigation pathways, complex menu options, excessive information, and difficulties with text entry. One particular feature of the DMHI evaluated in our previous study demanded users input text. This aspect proved to be challenging for most participants. The contributing factors included a small text entry field, minimized text size when entered, unfamiliarity with on-screen keyboards, lower literacy levels, and potential website bugs, such as misleading error messages indicating omitted entries when users had filled them in. Underscoring the importance of design that accounts for age, the same program was highly regarded as “straightforward” and “easy-to-use” when tested by research assistants, most of whom were in their 20s.

In terms of engagement, participants appreciated the characters and their stories, the digestible module format, and the in-session exercises throughout the sessions. However, some activities, especially those requiring considerable cognitive flexibility, posed a challenge for them. Regarding issues surrounding fit, some participants noted that it occasionally used complex or sophisticated language that was difficult to comprehend. A primary concern was the program’s lack of age-appropriate stories and case examples, leading to a perception that it was “not for someone like me.” Another recurrent complaint was the excessive length of some sessions. Despite being broken down into shorter segments or pages, these sessions sometimes required 2 to 3 hours or more, imposing a considerable burden on the users. In terms of knowledge, external support could enhance comprehension of session content. Finally, concerning implementation, participants welcomed the opportunity to apply the skills learned beyond the web-based sessions.

Based on the summary statistics of participants in Michigan’s 1915(c) MI Choice Waiver program, the typical profile of a low-income, homebound older adult is as follows: female (68%), White (75%), living alone (34%), aged between 65 and 79 years (36%), and experiencing diabetes (39%), and pain-related issues (44%).

Additional insights for the design were gleaned from semistructured interviews (n=14) and the web-based survey (n=17) conducted with social service agencies. At the client level, the primary barriers to DMHI implementation identified by social service providers were limited access to technology (n=17, 100%), low technology literacy (n=16, 94%), the stigma associated with mental illness (n=12, 71%), and cost constraints (n=10, 59%). Provider-level barriers included limited knowledge of geriatric depression, high caseloads, and competing demands. At the organizational level, potential barriers included a lack of financial incentives, reimbursement restrictions, and staff shortages.

### Design

The design of Empower@Home, based on insights from our elicitation phase, addressed the identified failure point with various features. We created a streamlined UI to enhance usability, incorporating intuitive navigation, clear call-to-action prompts (eg, “Press NEXT”), and large buttons, text, and print. Our design avoids complex menu options and information overload on any page, focusing on a responsive web layout where each page fits within a single screen of a 10-inch tablet or larger, thus eliminating the need for scrolling. Most exercises are implemented via a printed workbook, making on-screen text entry optional. The sessions are brief (20-25 min) and divided into short videos and occasional voice-over instructional pages. A video tutorial to familiarize users with the system and on-demand technical support is also available.

For engagement, our program uses video-based learning featuring diverse older adults and an overarching character-driven narrative featuring a homebound older adult named Jackie. The inclusion of in-session exercises throughout the sessions aims to maintain user involvement.

Regarding fit, we used plain language, age-appropriate case stories, and examples. Case stories and additional workbook information support knowledge acquisition. Finally, weekly home practices and modeling behavioral changes in the character-driven story are designed to aid the implementation of the CBT tools.

To further enhance our program, we introduced “Empower Coaches,” laypersons trained to provide weekly support calls to users, thereby addressing potential technical difficulties. This addition stemmed from user preferences for real-time support from a human over a fully automated system. Such external support is vital for populations with lower educational attainment and health or technology literacy, as it can bolster knowledge and implementation. While clinicians or therapists could fulfill the coaching role, we opted for laypersons or agency staff without specialized mental health training, such as caseworkers or community health workers. This decision considered the shortage of mental health professionals and the staffing structure of social service agencies serving older adults identified through our elicitation phase.

Stakeholder input influenced every aspect of the program design. To illustrate, input from older adult advisors informed the selection of the narrator’s voice, with a preference for lower-pitched voices with neutral American accents and slightly slower pacing. We also avoided using background music during voice-over narrations to prevent comprehension difficulties for those with age-related hearing loss [35]. Additionally, 1 group of social service providers identified the lack of diversity among inspirational quotes, leading to a more diverse selection in our program.

Jackie, the central character in the animated story series, was modeled on the typical profile of the Michigan’s MI Choice Waiver participants. Jackie is portrayed as a 74-year-old White female living by herself, similar to the typical participant profile. She also shares their health challenges, specifically diabetes and arthritis-related pain. The decision to animate the Jackie story was informed by small group discussions, in which older adult advisors unanimously preferred animated story series over those performed by actors. Animation also provided an opportunity to incorporate visual storytelling elements that deepened the Jackie narrative without overrelying on lengthy narration or dialogue. When the visual design of characters was presented to stakeholders for feedback, a strong preference was shown for designs that did not rely on stereotyped representations of older people as disabled or frail. The initial character designs were revised based on additional stakeholder feedback. Multimedia Appendix 2 shows example video frames from various episodes of the Jackie story.

### Development Cost

Excluding research staff time, replicators can expect a platform development cost near US $20,000 and monthly maintenance costs of around US $100. Our initial intervention development cost was US $10,125, supplemented by US $8700 for iterative refinements, totaling US $18,825. Regular upkeep, including server hosting with 2 central processing units, 4 GB RAM, 50 GB storage, and automated backups costs US $37 per month, plus US $50 per month for a dedicated database that is compliant with the Health Insurance Portability and Accountability Act. The University of Michigan Information and Technology Services provides the hosting service.

Additional expenses to consider are content creation costs, which can significantly differ based on creative requirements and chosen vendors. In our case, the animated story series “Jackie” cost US $32,900 to produce. The storyline, crafted by a freelance writer with a master of social work degree, incurred a cost of US $3000. In addition, voice-over recordings, performed by Fiverr-sourced artists who took on the roles of narrator, mindfulness exercise guide, and characters from the animated story series, added US $5000 to our expenses, making the total cost for the animated story series US $40,900.

### Usability Testing

#### In-Home Comparative Usability Evaluation

The in-home visits lasted between 90 and 120 minutes. Table 2 shows descriptive statistics of the 10 participants from the in-home usability evaluation. They were aged 71.4 years on average, and primarily identified as female (n=6, 60%). In total, 8 had at least a college degree (80%), and 5 (50%) had a household annual income of over US $50,000. They all owned a laptop or a computer and had internet access at home. All agreed or strongly agreed that they felt confident working on computers.

**Table 2:** Descriptive statistics of the in-home comparative usability study participants (N=10).

Characteristics		Values
Age (years), mean (SD)		71.4 (6.4)
**Gender, n (%)**		
	Male	4 (40)
	Female	6 (60)
**Race or ethnicity, n (%)**		
	Non-Hispanic White	9 (90)
	Hispanic	1 (10)
**Education, n (%)**		
	Some college, no degree	2 (20)
	Bachelor’s degree	3 (30)
	Graduate degree	5 (50)
**Marital status, n (%)**		
	Married or partnered	3 (30)
	Divorced or separated	3 (30)
	Widowed	3 (30)
	Never married	1 (10)
**Household income (US** $**), n (%)**		
	$10,000-$20,000	1 (10)
	$20,001-$30,000	1 (10)
	$30,001-$40,000	1 (10)
	$40,001-$50,000	2 (20)
	$50,001+	5 (50)
**Regularly used devices, n (%)**		
	Tablet or iPad	8 (80)
	Laptop or computer	10 (100)
	Smartphone	9 (90)

Table 3 shows the SUS scores by program tested. Normality tests were conducted to check the distribution of SUS scores, using the Stata commands *swilk* (the Shapiro-Wilk test for normality) and *sktest* (for skewness and kurtosis). All tests resulted in *P* values exceeding .05. These results failed to reject the null hypothesis, suggesting that the SUS scores followed a normal distribution. The average SUS score was 78 for Empower@Home, 55.8 for Beating the Blues, and 57.5 for MoodGym. SUS scores for Empower@Home had the smallest range and SD (score 78, SD 7.4), suggesting consistency in perceived usability across participants. In contrast, the SUS scores for Beating the Blues (55.8, SD 24.4) and MoodGym (57.5, SD 20.1) had large ranges and SDs. Results from paired 2-tailed *t* tests showed a significantly higher SUS score for Empower@Home compared to Beating the Blues and MoodGym, suggesting the superior perceived usability of Empower@Home over the 2 comparable programs.

**Table 3:** Usability statistics by programs tested during in-home evaluation.

Program tested	System Usability Scale^a^ score			
	Mean (SD)	Range (min-max)	Paired 2-tailed *t* test^b^ (df)	*P*(T>t) values
Empower@Home	78.0 (7.4)	65-87.5	Comparator	Comparator
Beating the Blues	55.8 (24.4)	2.5-87.5	*t*=3.28 (9)	.005
MoodGym	57.5 (20.1)	22.5-90	*t*=2.78 (9)	.011

^a^Higher total score indicates better usability.

^b^Paired 2-tailed *t* test compared the total SUS scores between Beating the Blues and Empower@Home, and between MoodGym and Empower@Home. Applying the Bonferroni correction, a 1-tailed *P* value of <.025 is statistically significant.

Of the 10 participants, 80% (n=8) preferred Empower@Home, reporting that they liked the mix of audio, video, and visual materials, and reported it being easy to use and engaging. Half of the users liked the narration and the animated story and said the story felt “real.” Most users felt that the look and feel of Empower@Home was neutral and had a clear layout. Two participants preferred Beating the Blues, liking its pacing, use of real actors, case examples, and in-session exercises. However, most participants, including the 2 who preferred Beating the Blues, reported that it was difficult to navigate its homepage, densely presented information, long loading time, and distracting “Urgent Support” button. Most users preferred a mix of audio, video, and visual materials, which were present in both Empower@Home and Beating the Blues. Most participants did not favor MoodGym for being text-heavy and having poor readability (small font and occasionally confusing terms or jargon). One participant shared a positive impression of MoodGym and liked it because they loved reading (however, this participant chose Empower@Home as their favorite).

Usability problems were found, particularly with the touch registration of the “Back” and “Next” buttons. The buttons, created with HTML’s <div> tag, function like hyperlinks requiring close pressing to the text. This issue is accentuated in older adults unfamiliar with touchscreens, who often apply long, hard presses, which capacitive touchscreens might not recognize. To address this issue, we replaced the <div> with the <button> tag to create an actual button that allows the entire button area to be clickable. Second, we implemented a dual color scheme to signal when a click command is registered. Third, we provided tips on interacting with a touchscreen in a short navigation tutorial played at the beginning of the program. Finally, we offered a stylus pen to participants to reduce problems caused by dry fingertips.

#### Longitudinal Field Testing

Four participants provided posttest data for the longitudinal field testing. They were all low-income homebound older adults enrolled in the Medicaid MI Choice Waiver program. Table 4 shows descriptive statistics of the participants. None of the 4 participants had a 4-year college degree. Two used the program on their own devices, and the other 2 used a 10.5-inch tablet provided by the study team. All participants had elevated depressive symptoms on the PHQ-9 before the start of the program (mean 12.75, SD 3.6).

**Table 4:** Descriptive statistics of the longitudinal field testing (n=4).

			Values
Age (years), mean (SD)			64.3 (3.4)
**Gender, n (%)**			
	Male	1 (25)	
	Female	3 (75)	
**Race or ethnicity, n (%)**			
	Non-Hispanic White	4 (100)	
	Hispanic	0 (0)	
**Education, n (%)**			
	High school	2 (50)	
	Some college, no degree	2 (50)	
**Marital status, n (%)**			
	Married or partnered	2 (50)	
	Divorced or separated	1 (25)	
	Never married	1 (25)	
**Household income (US $), n (%)**			
	$10,000-$20,000	2 (50)	
	$20,001-$30,000	2 (50)	
**Device ownership, n (%)**			
	Tablet, iPad, laptop, or computer	2 (50)	
	No device ownership	2 (50)	
Pretreatment PHQ-9^a^ score, mean (SD)			12.75 (3.6)

^a^PHQ-9: Patient Health Questionnaire-9.

**Figure 1:**
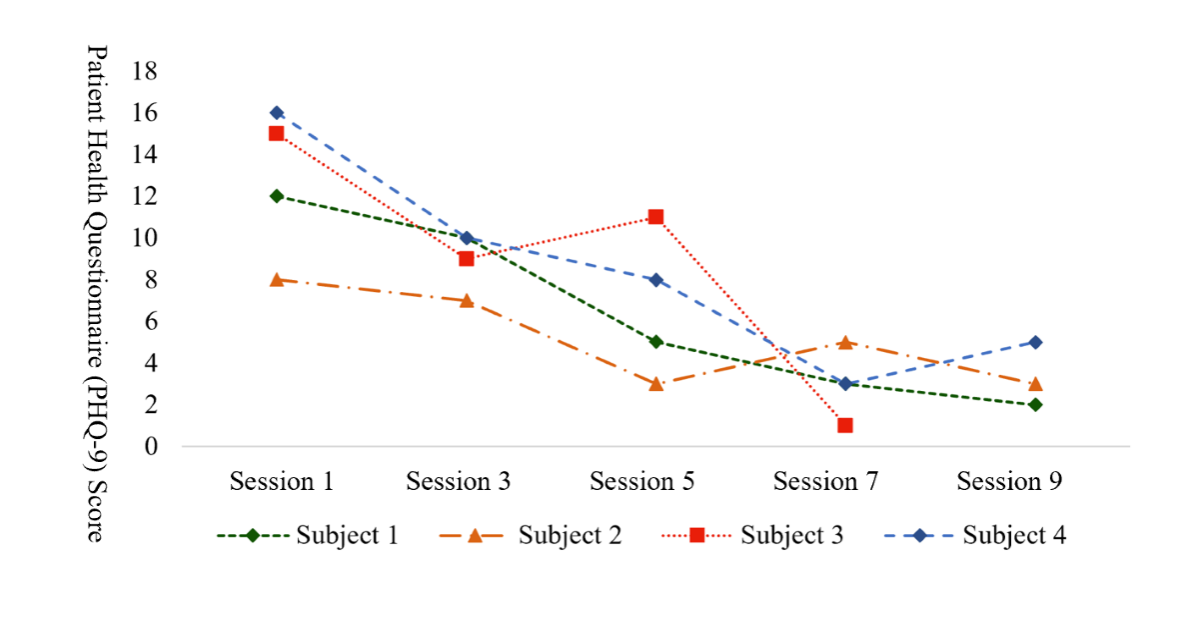
Single-subject line graph showing Patient Health Questionnaire-9 scores from in-app assessments.

At the end of the 10 weeks, 3 participants completed all 9 sessions, and 1 completed 8 sessions, suggesting excellent adherence rates. All participants agreed or strongly agreed that they liked the procedures used in this program and felt confident in their ability to perform the tasks required to participate in this program. The single-subject line graph (Figure 1) shows an overall trend of decreasing PHQ-9 scores over time.

## Discussion

### Principal Findings

Through a collaborative design process involving various stakeholders, we developed a DMHI incorporating CBT principles, age-related themes, engaging content, and an accessible UI. The in-home comparative usability evaluation results suggested that Empower@Home had higher perceived usability than Beating the Blues and MoodGym, 2 established iCBT programs. Most participants preferred Empower@Home over the other programs, citing its engaging multimedia content, clear layout, and relatable animated story. The longitudinal field testing results showed that low-income homebound older adults could adhere to the program with minimal support, suggesting the potential feasibility of the intervention.

Although the benefits of involving stakeholders in designing eHealth interventions, such as enhanced acceptability and engagement, are well discussed and acknowledged [36], members of some social groups continue to be excluded from full participation in the digital health ecosystem [37]. One such group is homebound older adults, who experience multiple social vulnerabilities and have limited technology literacy. Working with older adults with varying needs and technology literacy levels, we identified and addressed potential usability issues, such as touchscreen navigation difficulties, by refining the design and providing additional support and guidance to users. The program’s character-driven story was also developed in close collaboration with older adult advisors and drawing on the profile of low-income homebound older adults to ensure that the central character, Jackie, was representative of the population likely to receive the program as part of routine practice.

The COVID-19 pandemic has accelerated the use of web-based applications across multiple areas of health care, including mental health services [38]. The trend toward using DMHIs as part of routine care for those seeking treatment for mental health concerns is expected to continue. DMHIs that are cost-effective, scalable, ecologically responsive, and tailored have the potential to significantly expand treatment access, improve treatment outcomes, and support equity in mental health care. Researchers and clinicians developing DMHIs can learn from our experiences, which included close collaboration with community agencies and care recipients, an iterative design process, and close attention to user experience.

### Limitations

Although our results are promising, there are some limitations to consider. First, participants of the in-home usability evaluation were predominately non-Hispanic White and college-educated, which may not represent those likely to receive Empower@Home as part of routine practice. This may have resulted in overlooking UI challenges faced by other groups. Additionally, the in-home comparative usability evaluation involved a single session with each program, which may not fully capture the user experience throughout the intervention. Furthermore, although our longitudinal field testing was conducted with chronically ill, low-income homebound older adults, the sample size was small and lacked diversity. As our development process continues, we will continue to integrate feedback from participants from more diverse backgrounds and determine the efficacy of the intervention [39,40]. While we proactively considered implementation challenges during the design phase, future studies should systematically investigate implementation. Issues like coaching training and fidelity require further exploration to ensure the intervention is delivered as intended.

### Conclusions

In conclusion, the development of Empower@Home provides a valuable example of how DMHIs can be designed and developed through close collaboration with stakeholders, iterative design processes, and attention to user experience. DMHIs have the potential to significantly expand access to mental health care and improve treatment outcomes, and future research should continue to explore the efficacy and implementation of these interventions.

## Appendix

Multimedia Appendix 1 Empower@Home session content example web pages. From left to right: video, text with voice-over, and mood self-check score page.

Multimedia Appendix 2 Example video frames from the animated story of Jackie.

Multimedia Appendix 3 Empower@Home user workbook example pages. From left to right: session summary, in-session exercise, home practice, and inspirational quote.

Multimedia Appendix 4 Empower@Home user interface example pages. From left to right: program homepage, video page, and provider dashboard.

## Abbreviations

CBT: cognitive behavioral therapy
DMHI: digital mental health intervention
iCBT: internet-based cognitive behavioral therapy
PHQ-9: Patient Health Questionnaire-9
SUS: System Usability Scale
UI: user interface
