# Supplementary material for: Web-Based Cognitive Behavioral Therapy for Depression Among Homebound Older Adults: Development and Usability Study
Source: JMIR Aging. 2023 Sep 19;6:e47691. doi: 10.2196/47691 (PMC10548322; doi:10.2196/47691)

Empower@Home session content example web pages from left to right: video, text with voice-over, and mood self-check score page.


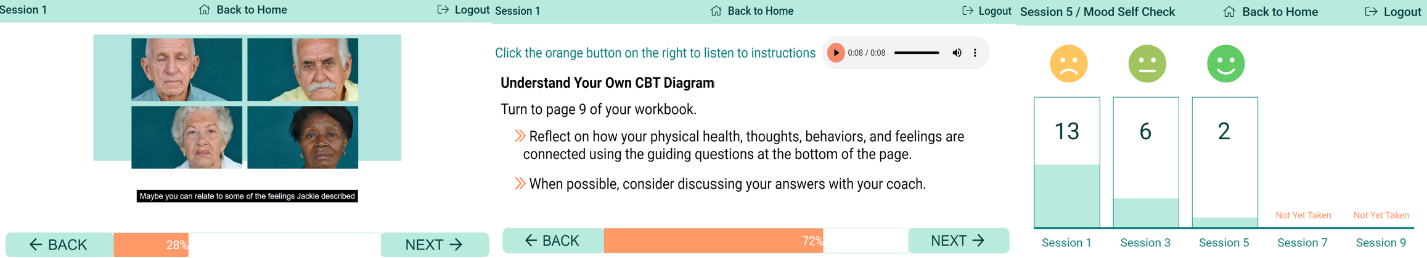

Supplement: Multimedia Appendix 1 [file aging_v6i1e47691_app1.docx]
