# Supplementary material for: Web-Based Cognitive Behavioral Therapy for Depression Among Homebound Older Adults: Development and Usability Study
Source: JMIR Aging. 2023 Sep 19;6:e47691. doi: 10.2196/47691 (PMC10548322; doi:10.2196/47691)
Supplement: Multimedia Appendix 3 [file aging_v6i1e47691_app3.docx]

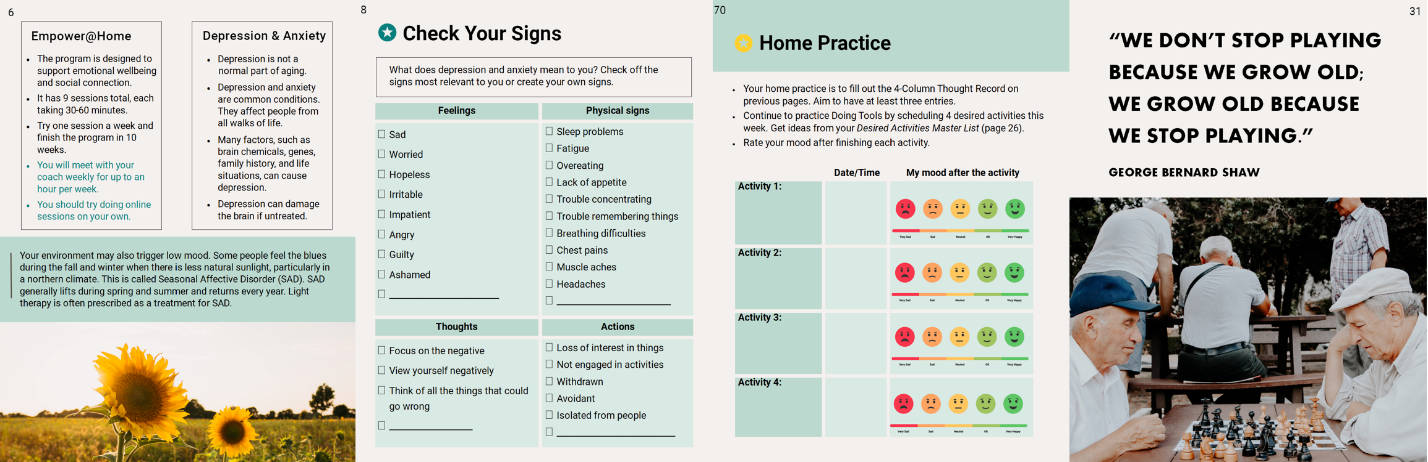


Empower@Home user workbook example pages from left to right: session summary, in-session exercise, home practice, and inspirational quote.
